# Draft Genome Sequence of Clostridium senegalense Strain AGRFS4, Isolated from a Dairy Farm in New Zealand

**DOI:** 10.1128/MRA.00214-20

**Published:** 2020-05-07

**Authors:** Tanushree B. Gupta, Ruy Jauregui, Amila Srilal Nawarathana, Gale Brightwell, Paul Maclean

**Affiliations:** a Food Assurance Team, Hopkirk Research Institute, AgResearch, Palmerston North, New Zealand; b Knowledge and Analytics, Grasslands Research Centre, AgResearch, Palmerston North, New Zealand; University of Maryland School of Medicine

## Abstract

We report the draft genome sequence of a new Clostridium senegalense strain, AGRFS4, which was isolated from a dairy farm environment in the Manawatu region in New Zealand. The genome is 3.98 Mb, with a GC content of 27%. The genome sequence was found to be 86.6% similar to that of the type strain Clostridium senegalense JC122. Until now, no Clostridium senegalense strain from New Zealand has been reported.

## ANNOUNCEMENT

The genus *Clostridium* consists of more than 180 species of obligate and facultative anaerobic rod-shaped bacilli that are capable of producing highly heat- and chemical-resistant endospores (1, 2). While some species, such as Clostridium botulinum, Clostridium difficile, Clostridium tetani, and Clostridium perfringens, are known pathogens (1, 3–6), Clostridium butyricum can be used as a probiotic (7, 8).

Clostridium senegalense JC122 is a type strain that was first isolated from a stool sample from a healthy male subject in Dielmo, Senegal; its draft genome sequence was published in 2012 (9). Here, we report the whole-genome sequence of a new C. senegalense strain, AGRFS4, which was isolated from a soil sample from a New Zealand dairy farm. The sequences obtained will be used to investigate any pathogenic or beneficial traits of this isolate.

Bacteria were isolated using previously described methods (10). Briefly, 10 g of soil was suspended in 50 ml of phosphate buffer (PB) and centrifuged at 3,466 × *g* for 1 h. The pellet was resuspended in 5 ml of PB and heated at 80°C for 10 min. One milliliter of the heated sample was added to cooked meat-glucose-starch medium (11) and incubated anaerobically at 35°C for 48 h. The growth suspension was serially diluted, plated on Shahidi-Ferguson agar (12), and incubated anaerobically for 24 h to yield pure colonies. Genomic DNA was extracted from the pure cultures grown in tryptic soy broth (Fort Richard Laboratories, New Zealand) by using a phenol-chloroform extraction method (13). The quality and concentration of DNA were determined using a Qubit 2.0 fluorometer (Thermo Fisher Scientific, USA). Initial identification was conducted using 16S rRNA amplicon sequencing with the forward primer pA (5′-AGAGTTTGATCCTGGCTCAG-3′) and the reverse primer pH* (5′-AAGGAGGTGATCCAGCCGCA-3′) (14). The amplification method consisted of 93°C for 3 min; 92°C for 1 min, 55°C for 1 min, and 72°C for 2 min for 30 cycles; and a final extension at 72°C for 3 min.

The whole genome of C. senegalense strain AGRFS4 was prepared with the NuGEN Celero PCR workflow with enzymatic fragmentation DNA library preparation kit and sequenced using the Illumina MiSeq version 3 sequencing platform (Massey Genome Services, Palmerston North, New Zealand) to produce 500,848 read pairs of 300 nucleotides and 301,510,496 bp, giving ∼75-fold coverage. The reads were quality trimmed, filtered, and assembled via the A5-miseq pipeline version 20160825 with default settings (15). The assembly produced 95 contigs with a total genome size of 3.98 Mb, an *N*_50_ value of 14 kb, and a GC content of 27%. A BUSCO version 3.0.2 (16) test using the bacterial reference produced a completeness score of 98%.

Analysis of 16S rRNA sequencing data showed 98.2% sequence similarity between C. senegalense AGRFS4 and C. senegalense JC122^T^. A comparative genomic analysis was performed with the genome sequences of these organisms using the Type (strain) Genome Server (TYGS) (https://tygs.dsmz.de). *In silico* digital DNA-DNA hybridization (dDDH) was used to calculate genome-to-genome distances (17), which resulted in a dDDH (d_6_) value of 86.6%, indicating the same species but with probable differences at the strain level. Further studies are required to investigate these differences.

### Data availability.

The raw reads have been deposited in the NCBI database under accession number PRJNA605262. This whole-genome shotgun project has been deposited in DDBJ/ENA/GenBank under accession number JAAGPU000000000.
